# Improvisation and the self-organization of multiple musical bodies

**DOI:** 10.3389/fpsyg.2015.00313

**Published:** 2015-04-20

**Authors:** Ashley E. Walton, Michael J. Richardson, Peter Langland-Hassan, Anthony Chemero

**Affiliations:** ^1^ Department of Psychology, Center for Cognition, Action and Perception, University of CincinnatiCincinnati, OH, USA; ^2^ Department of Philosophy, University of CincinnatiCincinnati, OH, USA

**Keywords:** music improvisation, self-organization, movement coordination, complex dynamical systems, multiscale analysis

## Abstract

Understanding everyday behavior relies heavily upon understanding our ability to improvise, how we are able to continuously anticipate and adapt in order to coordinate with our environment and others. Here we consider the ability of musicians to improvise, where they must spontaneously coordinate their actions with co-performers in order to produce novel musical expressions. Investigations of this behavior have traditionally focused on describing the organization of cognitive structures. The focus, here, however, is on the ability of the time-evolving patterns of inter-musician movement coordination as revealed by the mathematical tools of complex dynamical systems to provide a new understanding of what potentiates the novelty of spontaneous musical action. We demonstrate this approach through the application of cross wavelet spectral analysis, which isolates the strength and patterning of the behavioral coordination that occurs between improvising musicians across a range of nested time-scales. Revealing the sophistication of the previously unexplored dynamics of movement coordination between improvising musicians is an important step toward understanding how creative musical expressions emerge from the spontaneous coordination of multiple musical bodies.

“Human beings learn and do things that have never been done before."

(Smith, 2010, p. 344)

On a first reading, Linda Smith’s statement does not seem particularly earth shattering. Yet the novelty and creativity of human behavior is precisely what makes it exciting and difficult to understand, as well as difficult to replicate in terms of artificial intelligence. These abilities are not limited to genius inventors or idiot savants; they are used in any instance in which a task is carried out without a detailed plan of execution, where the steps and goals are not predetermined, but discovered through the course of action (Smith, 2010). Within the research literature this ability is discussed using terms like creativity studies (Duncker, 1945), abductive reasoning (Kloos and Van Orden, 2012), emergence and insight problems (Ohlsson, 2011). And while the focus of these studies may range from the processes engaged by Van Gogh when creating a master painting (Ghiselin, 1952), to observing how participants fasten a candle to a door with limited and misleading materials (Duncker, 1945), even everyday, planned tasks require constant moment-to-moment improvisation. You can never step into the same river twice, never play the exact same game of soccer, never navigate your car through the exact same highway traffic, or cook your favorite meal the exact same way – “life is a continual improvisation” (Agre and Chapman, 1987, p. 268).

Given the challenges in understanding the processes behind everyday behaviors and tasks, explicating the skills behind musical improvisation is vastly more complex. Explanations of this ability thus far have focused on exactly how musicians are able to engage in performance that demands immediate, yet coherent musical expression from an infinite number of combinatorial possibilities. Pressing (1998, 2000) and Berkowitz (2010) both developed theories for how musicians can engage in such spontaneous musical action. Pressing focuses on the skills and tools musicians use to overcome the limitations of their information-processing capacities. Berkowitz proposes a cognitively economical “knowledge base” which optimizes speed and efficiency by organizing musical materials into higher-level categories according to their function (Berkowitz, 2010, p. 54). This knowledge base is refined through practicing variants of different musical expressions, as well as developing an understanding of how these units can be combined appropriately – a skill he calls recombination. This skill is achieved through statistical learning, where transitional probabilities that describe the likelihood of one musical event following another form patterns, which are captured in the hierarchical structure of the musician’s knowledge base. This is meant to ameliorate the overwhelming task of choosing from the infinite possibilities such that when an improviser anticipates the need for a certain musical phrase, the knowledge base is drawn upon and “the ears and/or body” can “choose a variant suitable to the musical situation at hand” (Berkowitz, 2010, p. 54).

While fully appreciating the complexity of musical improvisation can seem to render its explanation impossible, this paper will attempt to outline a new approach to clarifying how this behavior is achieved, and how, as Berkowitz (2010) says, the ears and the body choose. The first section will discuss the role of body movement in music performance as well as the perception of musical action. The second section will move forward from the role of body movement in communication between a musician and a passive listener, to consider the complexities of movement coordination in the communication between two active co-performers. The third and fourth sections will introduce new methodological and theoretical approaches that we argue can help address the complexity of inter-musician movement coordination by uncovering the dynamics of the non-stationary, aperiodic and spontaneous behavioral coordination that characterizes musical improvisation.

## The Lives of Bodies

Not only is the act of musical improvisation demanding and complex, but at the peak of performance musicians are often unaware of their actions and the details of how their skills are being executed. Berkowitz (2010, p.125) calls this the “creator-witness phenomenon”; Jeff Pressing explains how “... the hands appear to have a life of their own, ... in a sense, the performer is played by the music” (Pressing, 2000, p. 139). Not only is this experience common to improvising musicians, but this sense of “flow” is considered to be particularly important to higher quality performance as well as increased psychological well-being (Csikszentmihalyi, 1990; Csikszentmihalyi and Csikszentmihalyi, 1992; Croom, 2012). Berkowitz claims that “letting go” allows for the automated components and processes that make up a musician’s expert knowledge base to run the show. But despite its importance, exactly how this knowledge base and these processes are at play during these creator-witness experiences is left an enchanting mystery, where the “musical flow *magically* [emphasis added] manifests, without a need to know or remember where one has been or where one is going” (Berkowitz, 2010, p. 130).

In these moments of limited conscious awareness and heightened creativity, the same statistical regularities and transitional probabilities Berkowitz describes as being encapsulated by this conceptual knowledge base, map directly onto sensory-motor experiences (Smith, 2010). Focusing on an explanation of how a stable knowledge base supports improvisational musical expression forgets the lives of the hands (Sudnow, 1978), the choices of the ears (Berkowitz, 2010)– the body’s own actions (Berliner, 1994). As we will explain, this widespread attribution of agency to the body and its limbs goes beyond simple metaphor to provide new possibilities for discovering stable patterns in the behavior of improvised performance, aside from a conceptual knowledge base.

The importance of body movement in understanding musical performance has become well accepted, championed by Vijay Iyer in his detailed accounts of how musical bodies tell stories (Iyer, 2002, 2004). The dynamics of movement and force in musical performance have been widely examined experimentally, (see Keller, 2012; Palmer, 2013 for review), with kinesthetic patterns found to be the primary determinant of everything from musical genres, to structures of instruments, as well as musician’s personal identities (Baily, 1985; Dalla Bella and Palmer, 2011). This bodily motion is essential to understanding not only the production but also the perception of music. Listeners directly experience the “articulators” of performers, hearing the changes in rate and force of their body movements that produce musical sounds (Shove and Repp, 1995). Thompson et al. (2005) reviewed evidence for how visual information about performer’s movements influences listeners’ perception of music, examining the bodily-mediation of facial expressions and limb gestures used by musicians to highlight and articulate phrases within the performance. These kinesthetic “affect displays” of performers like BB King opening his mouth and shaking his head to match the vibrato of a note, serve to constrain the perceptual experience of listeners (Thompson et al., 2005, p. 207). These musical movements are not considered peripheral, but as providing direct access to the perceptions, moods and feelings of listeners (Maes et al., 2014).

Thus the kinesthetic dimension is crucial to investigating the complexities of music improvisation. And with the discussion of bodily mediation as well as bodily actions, we see the importance of body movement both in the production of sonic events, and in the communicative processes that make up a musical performance. Understanding music improvisation is not only about the brain and the body, but also the environment: an environment that can include an audience as well as other performers. Given the already long list of demands on musicians when improvising, it seems that the additional task of communication makes the ability seem even more “magical,” and explicating the processes behind “letting go” more elusive. Musicians not only have to spontaneously produce innovative and cohesive musical expressions from an overwhelming number of combinatorial possibilities, but simultaneously interpret and coordinate with those produced by other performers. Nardone (1997), in interviews with musicians about how they improvise, uncovers the tactic of “ensuring spontaneity while yielding to it” (Berkowitz, 2010, p. 125). Examination of these communicative, bodily processes can provide a way of understanding exactly what is happening when musicians “let go” and submit to musical spontaneity.

## Spontaneous Coordination of Multiple Musical Bodies

There is still much left to understand about the complexity of the coordination and communication that occurs between groups of improvising musicians, especially because many of the experimental investigations thus far have focused on individual improvisers (e.g., Keller et al., 2011; Norgaard, 2011, 2014). Yet the paradigmatic example of improvisation is a jazz quartet, where multiple musical bodies must spontaneously coordinate while simultaneously engaging in both musical perception and action. They engage in a continuous negotiation– anticipating and coordinating their playing behavior without the guide of musical notation. Musical performance emerges within a context of social collaboration, resulting from the ongoing interactions among multiple individuals, where members are collaborating to construct and negotiate the flow of the performance from moment-to-moment (Sawyer, 2003).

Understanding the dynamics of the performance of musical groups will not be achieved by linear decomposition, isolating out an independent flow of each individual musician’s conceptual knowledge base or musical movements. Interpersonal coordination is not easily isolated into components defined by content or a particular frame of time. Musician’s movements may at times involve explicit communicative signals such as a touch to the head that signals “back to the top,” or eye contact and nodding of the head before or after solos. But these are just a small part of a continuous flow of information about a co-performer that supports adaptive coordination and communication across the multiple time scales of an improvised musical performance. When engaged in the continuous action and perception required of musical coordination, it is not possible to identify the different component parts of a co-improvisers’ actions and their exact intended meaning within the flow of performance.

The perceptual delays and latencies inherent to group performance present another challenge to investigating the continuous nature of spontaneous musical coordination, whether it is a duet, quartet or orchestra (Davies et al., 2013). And while these delays are measured in milliseconds, in some instances certain musical forms like jazz are dependent on deviations from the beat at very small time scales. And these deviations have not only been found to be preferred by listeners (Hennig et al., 2012), but even to be an index of the health of a musician’s physical system (Ruiz et al., 2014). The demands on musicians are mounting: they must choose from an infinite set of possible musical expressions, continuously adapting and coordinating their musical action and communication with co-performers, despite perceptual delays, in order to produce something meaningful within the context of the performance.

Understanding the dynamics of this improvisatory behavioral coordination is not trivial. Continuous adaptation is also demanded of groups of musicians performing a pre-rehearsed piece (Keller, 2008), but the lack of structure in improvisational performance potentiates anticipatory coordination that can result in dramatic transitions toward unexpected trajectories. Much of the experimental work that investigates groups of musicians’ movements has examined the dynamics when musicians play a pre-rehearsed musical score (Keller and Appel, 2010; Loehr and Palmer, 2011; Palmer and Loehr, 2013; Ragert et al., 2013). Yet the unstructured nature of improvisation is such that these new trajectories are discovered when musicians act upon information that they detect about their co-performer, as well as adapt their playing in order to re-contextualize and take advantage of musical errors or “noise.” Anticipatory movement coordination or musical motor predictions (Novembre and Keller, 2011) are an important part of the initiation of these transitions to novel modes of expression. Even unintended fluctuations in performance are crucial to the development of new musical structures (Borgo, 2005), and there is reason to believe that these fluctuations can occur in the kinesthetic as well as the sonic dimension. As saxophonist Evan Parker puts it, “sometimes the body leads the imagination”(Borgo, 2005, p. 58).

Quantifying the collective changes and transformations in the body movement coordination between improvising musicians can help explicate the complexity of spontaneous performance, as well as provide a glimpse of the dynamics that make possible the emergence of previously unimagined forms of order. In the next section we introduce methods for quantifying the evolution of inter-musician movement coordination continuously across the time span of a performance, capturing the behavior at multiple, nested time scales.

## Measuring Spatiotemporal Patterns of Musical Spontaneity

“*The major role of improvisation in many oral musical traditions, combined with the important function of groove, make possible alternative notions of musical form … in which meaning of music is located in the free play of smaller constituents*.”

(Iyer, 2008, p. 278)

In trying to understand the complexity of generating new musical meaning in improvisation, many describe the processes by which musicians learn to spontaneously combine smaller sonic units. Berkowitz (2010) describes how musicians introduce variation to musical patterns at multiple levels, recombining smaller elements to form new musical entities. Yet we suggest that the behavioral coordination that occurs between improvising musicians may be best conceptualized as emergent, involving the continuous self-organization of the perception and action processes that support musical play. The tools of complex dynamical systems provide powerful methods for the investigation of both sonic and kinesthetic patterns at multiple time scales, while avoiding an “arbitrary segmentation” of the continuous flow of information for musical perception and action (Demos et al., 2014, p. 2). Complex dynamical systems are those that consist of a large number of interacting components that give rise to collective behavior that can be difficult to anticipate from knowledge of the individual components that make up the system (Richardson et al., 2014). Dynamical models have been employed in understanding how musicians improvise (Pressing, 1999, 2000) and coordinate with rhythmic sequences (Loehr et al., 2011), but David Borgo’s 2005 book *Sync or Swarm* is the most detailed account of exploring the skill of musical improvisation using the statistical tools of complex dynamical systems theory. More recent applications of these methods to examine musical movements and musical structure include: fractal analysis (Beauvois, 2007; Rankin et al., 2009; Demos et al., 2014; Hennig, 2014; Ruiz et al., 2014) recurrence quantification analysis (Serrà et al., 2009, 2011; Demos et al., 2011) and sample or Shannon entropy (Keller et al., 2011; Goldman, 2013; Glowinski et al., 2013).

Here we will focus specifically on the potential of cross wavelet spectral analysis for investigating musical movement coordination. Cross wavelet spectral analysis is a non-linear time series method that has been widely used in the fields of geological sciences and physiology. More recently Schmidt et al. (2014) has demonstrated its use in understanding the movement coordination that occurs between co-actors during joke-telling and dancing (Schmidt et al., 2014; Washburn et al., 2014). As noted by Schmidt et al. (2014), the advantage of cross wavelet analysis is its ability to reveal common periodicities in behavioral coordination at nested time scales, detecting local microscale structures (e.g., note or bar) within global macroscale patterns (e.g., chorus or piece). Importantly, it provides this local and global information without assuming the time series is stationary. This means it is able to capture the time-evolving behavior in time series that are noisy, contain a drift or sudden change in the mean, or a “brutal” change in frequency (Issartel et al., 2006, p. 151)– all of which are likely in the complex patterns of coordination that emerge during spontaneous musical performance.

**FIGURE 1:**
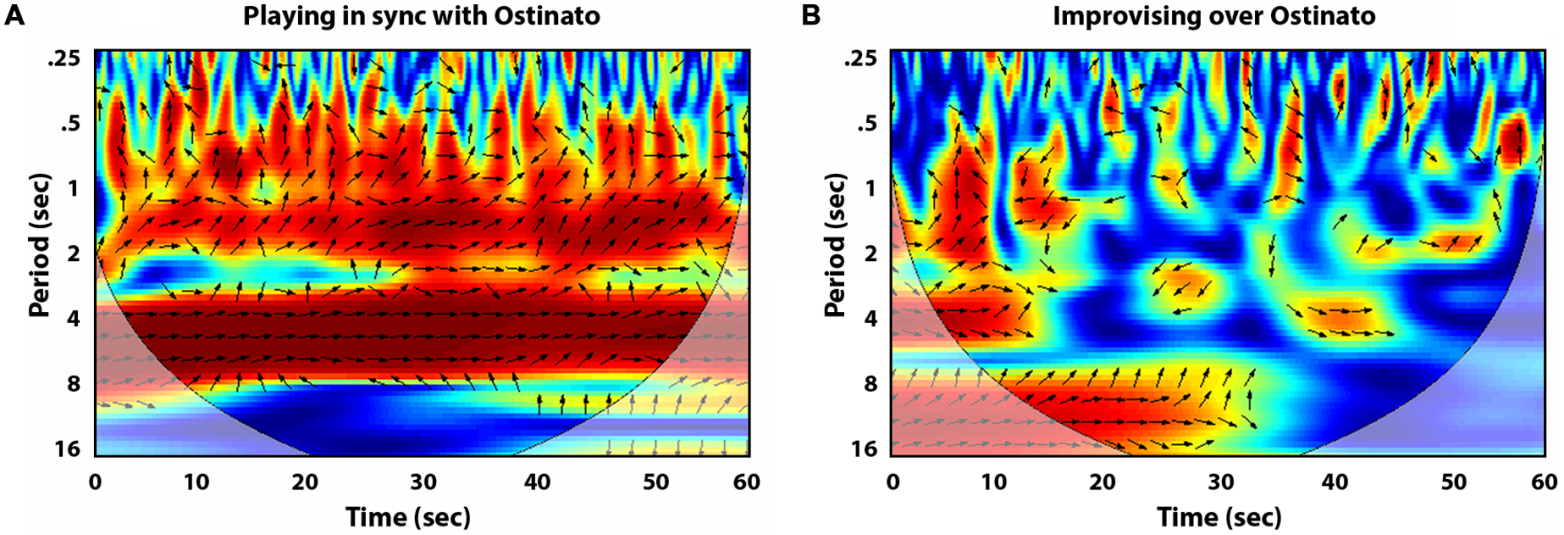
**Cross wavelet plots of the lateral movements of the musicians’ right forearms, displaying the strength of coherence at each period (red for high coherence = 1, dark blue for low to no coherence = 0), as well as relative phase angle (right arrows equal in-phase coordination, left arrows equal anti-phase coordination)**. **(A)** Displays the coordination between two piano players playing the exact same part, in synchrony with the *ostinato* backing track. **(B)** Displays coordination while the musicians improvise over the *ostinato* backing track.

**FIGURE 2:**
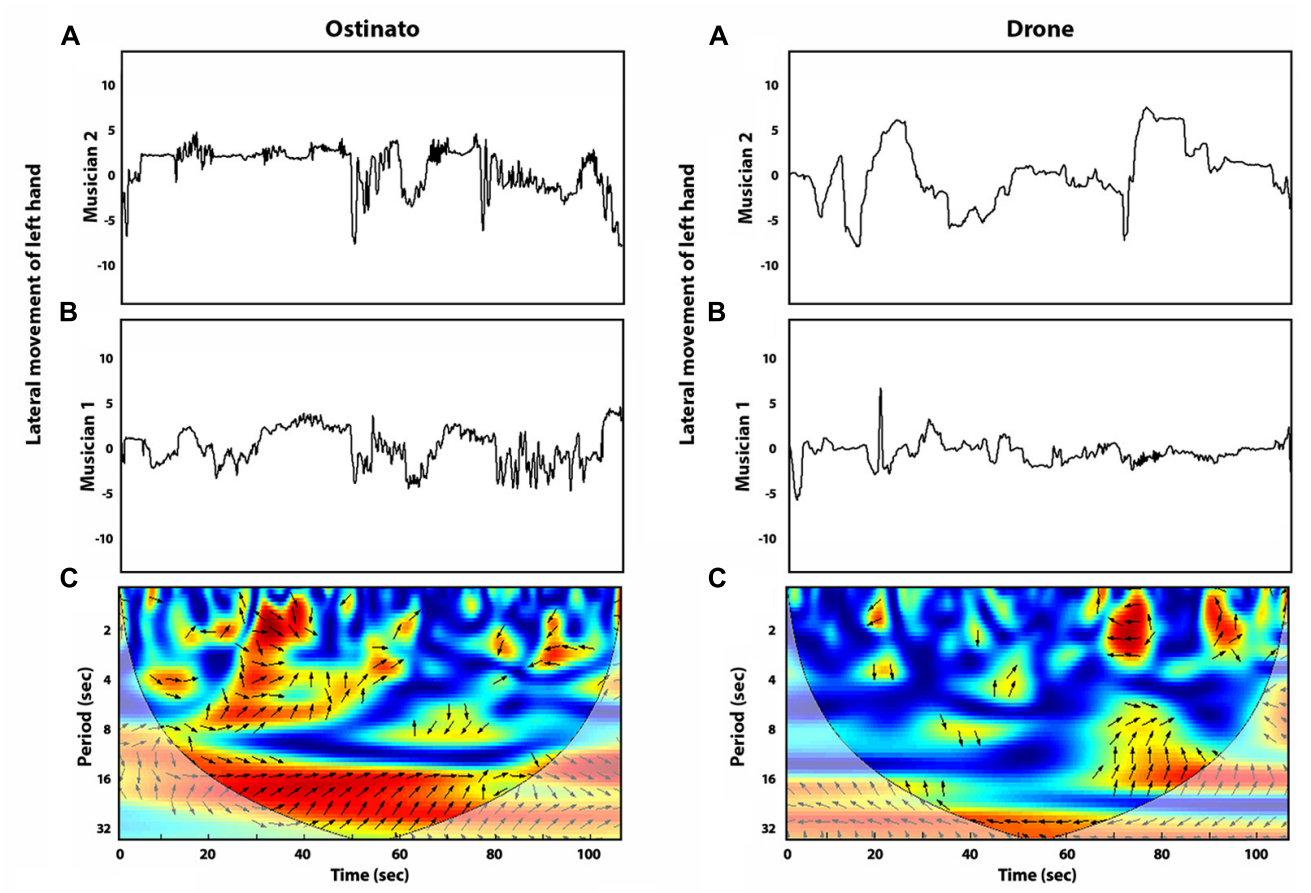
**Cross wavelet analysis of experimental data from two piano players improvising with an *ostinato* backing track (Left) and *drone* backing track (Right)**. **(A**,**B)** display the normalized time series of the lateral movements of the musicians’ left hands. **(C)** Cross wavelet analysis of these two time series displaying the strength of coherence at each period (red for high coherence, dark blue for low to no coherence), as well as relative phase angle (right arrows equal in-phase coordination, left arrows equal anti-phase coordination).

More specifically, cross wavelet analysis assesses coordination between two time series through spectral decomposition, and subsequent examination of the strength (coherence) and patterning (relative phase) of the coordination that occurs between participants across multiple time scales (see Grinsted et al., 2004; Issartel et al., 2014, for a more detailed introduction). The strength of coordination and the relative phase angle between two time series is assessed for shorter, half second and second-to-second time-scales, as well as at longer 4, 8, 12, and 16 s time-scales. **Figures 1** and **2** demonstrate the use of cross wavelet analysis to investigate the coordination between the lateral movements of the forearms of two piano players. Their movements were recorded using wireless motion-tracking sensors attached to their wrists while they improvised over a musical backing track. The time series of their limb movements were then analyzed using functions available in MathWorks’ free wavelet toolbox [Copyright (C) 2002-2004, Aslak Grinsted]. The movement time-series were also low-pass filtered prior to analysis using a 10 Hz Butterworth filter. For each of the different time scales an average measure of the correlation of the time series was calculated on a scale from 0 to 1, as well as the average distribution of relative phase angles (DRP) that occurred between the musicians’ movement time series. One can observe the strength of coherence over the course of the performance as denoted by color (red for high coherence, dark blue for low to no coherence) for each period (in units of seconds) on the *y*-axis. The arrows correspond to the relative phase of the coordination. Right arrows equal in-phase coordination (the two systems are visiting the same states in perfect synchrony) and left arrows equal anti-phase coordination (the two system are visiting states that are in perfect opposition).

One particular advantage of this analysis is the ability to determine how movement coordination relates to the shorter- and longer-term temporal structure and phrasing of the musical context. This is demonstrated by comparing the cross wavelet plots of the coordination that occurred between the lateral movements of pianists’ right forearms in (**Figures 1A,B**). **Figures 1A** displays the coordination that occurred between the pianists when instructed to play along to an *ostinato* backing track together in perfect synchrony. The *ostinato* backing track contains a melodic phrase consisting of four ascending chords (Cm11; BbM7/D, EbM7#11, Fadd4) that is repeated every 4 s. Accordingly, the cross wavelet plot reveals a high degree of coherence (i.e., red) and in-phase coordination (right pointing arrows) at the 4-second interval. **Figures 1B** is a cross wavelet plot of the right arm movement of the same musicians instructed to improvise with one another over the *ostinato* track. As one would expect, there is much less coherence and stable in-phase behavior. However, musicians still exhibit pockets of coordinated behavior, particularly at the spectral scale (*y*-axis) of 8 to 16-s. Because the 4-s melodic phrase in the *ostinato* track repeats four times (a total interval of 16 s) this indicates that the musicians treated this as a meaningful unit-interval and transitioned to new musical phrases at divisions of this temporal unit. That is, the musicians moved their hands so they could play new keys currently out of reach at this time-scale, thus initiating similar lateral hand movements across the keyboard.

This coordination can then be used to compare the dynamics that emerge as a function of the musical context: **Figure 2** (Left) shows the coordination of the musician’s left arms while improvising over the *ostinato* track, and **Figure 2** (Right) was captured while the musicians were improvising with a *drone* backing track, consisting of one pair of chords (D and A of second lowest octave) played continuously for the duration of the performance. The *drone* track as compared to the *ostinato* lacks melodic and rhythmic structure, thus these backing tracks represent two different levels of complexity in the musical context, constraining the musicians’ behavior in different ways. As a result they gave rise to different patterns of inter-musician movement coordination.

Capturing patterns of synchronization across multiple time scales of musical movements is useful not only in the case of the hand, but for the oscillatory motions of other limbs which may initially be considered more gestural or peripheral to musical production. In his book *The Ways of the Hand*, David Sudnow documents in detail his experience training and developing the ability to improvise jazz on the piano. He recognizes a key turning point in his skill development to be observing performances by the New York jazz piano player Jimmy Rowles. He describes:

“I watched him night after night, watched him move from chord to chord with a broadly swaying participation of his shoulders and entire torso, watched him delineate waves of movement, some broadly encircling, others subdividing the broadly undulating strokes with finer rotational movements… As his foot tapped up and down his head went through a similar rotational course, and the strict up-and-down tapping of the foot was incorporated in a cyclical manner of accenting his bodily movements. In an anchored heel you could see only the up-and-down movements of the foot, but in the accompanying head rotation and shoulder swaying you could see a circularly undulating flow of motion…”

Sudnow (1978, p. 82)

**FIGURE 3:**
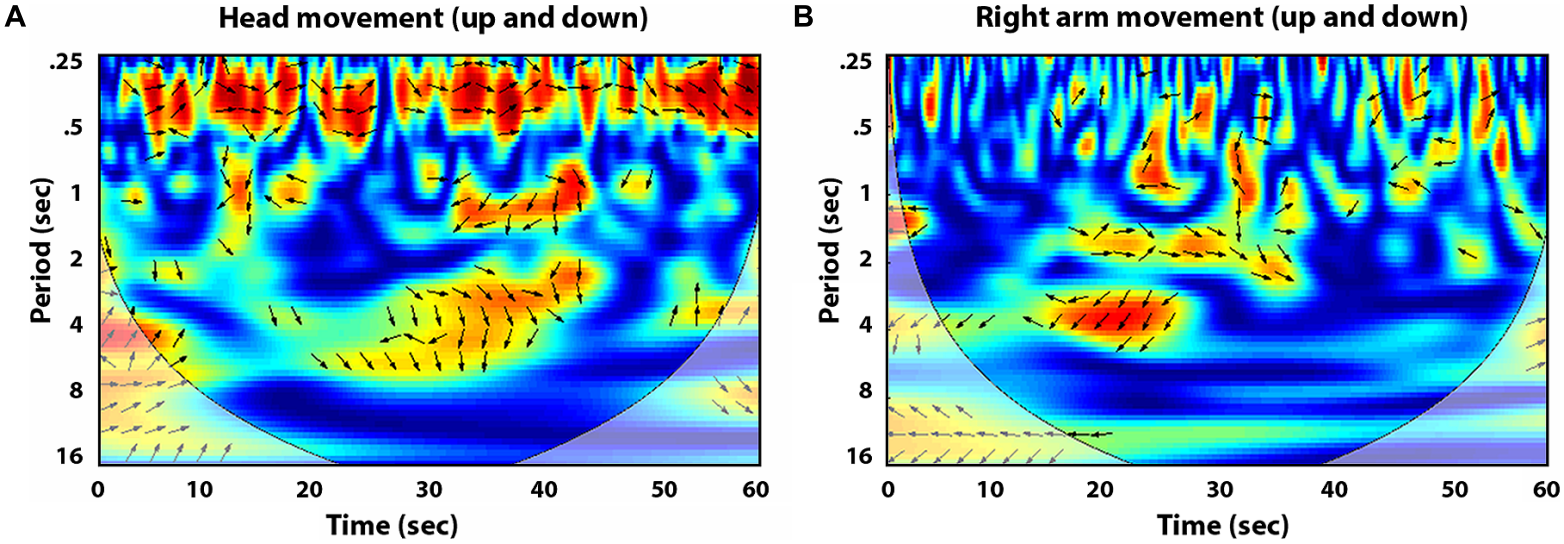
**Cross wavelet analysis of experimental data from two piano players improvising with a *swing* backing track**. **(A)** Displays coordination of the musician’s upward and downward head movements, while **(B)** displays the coordination of the upward and downward movements of the musicians’ right hands.

Sudnow (1978) goes on to explain how after observing the full spectrum of Jimmy Rowles’s bodily articulations, he was able imitate these dynamics in his own playing to produce similar musical qualities. Cross wavelet spectral analysis provides tools to take these eloquent descriptions of performers’ movements and evaluate quantitatively the relationships between the rotational movements of the different limbs – how they encircle and subdivide one another to produce the “undulating flow of motion” of musical production (Sudnow, 1978, p. 82). It can be used to explore differences in frequency and patterning of limb movements with relationship to their performative function: for example the left hand which plays more accompanying parts can be compared to the right hand which often engages in melodic leads, or the movement of the foot while tapping along with the beat as compared to when it is engaged in pressing a sustain pedal. **Figure 3** compares the results of a cross wavelet analysis performed on the coordination of musician’s up-and-down head movements (**Figures 3A**) with that of the up-and-down movements of their right forearms (**Figures 3B**). For this trial the musician’s head movements are more highly coordinated than the up-and-down movements of the hands, at a much faster time scale. While the movements of the hands may be considered more functionality relevant for musical production, velocity of head movements have been found to play a large role in performance expressivity (Castellano et al., 2008; Juchniewicz, 2008). Further exploring these dynamics can reveal the role this movement plays in how musicians spontaneously coordinate their playing behavior within improvised performance.

Non-linear time series methods make possible detailed examinations of how the dynamics of movement coordination between improvising musicians unfold across the time span of musical performance. Observing how and when stable patterns in these dynamics emerge and evolve provides new possibilities for exploring the skill of improvisation, as well what dynamics contribute to more successful musical performance. There is a large body of work dedicated to examining the brain activity that occurs during improvisation (Limb and Braun, 2008; Donnay et al., 2014; Beaty, 2015 for review), but as Sudnow (1978, p.146) explains, “to define jazz is to describe the body’s ways”– a body that is not only a brain. The role of the approach introduced here is to assure that experimental investigations do not miss out on the full spectrum of coordination and joint actions essential to musical communication, and to provide tools for capturing the way that musicians *interact with each other*. Notice, for example, focusing just on brain activity or note production would have missed the surprising coordination patterns found in head movements, and the ways that these differ from the coordination patterns of the musicians’ right hand movements. Observing how the dynamics evolve within the performance and how different manipulations of the performance context (i.e., backing track) change these dynamics, can help develop predictions about how within spontaneous musical exchanges performers can successfully anticipate and generate expectations for future musical events for each other to actualize.

## Self-Organization of Musical Meaning

When a jazz trio plays an improvisational piece their actions become so tightly coordinated and their decisions so seamlessly intertwined that the trio behaves as a single synergistic system rather than a collection of individuals. The principles of dynamical self-organization provide the language with which to describe the way performers exploit “the constraints and the allowances of the natural timescales of the body and the brain as a total physical system” (Iyer, 2008, p. 276). In live improvisation, performers must simultaneously be both producers and receivers of musical signs, the bodies of the musicians telling stories to their co-improvisers. That is, each individual improviser allows her activity to be constrained by the sonic and kinesthetic results of the activities of the other improviser. When coupled together during musical performance, their signal producing and signal receiving processes not only overlap, but serve as constraints on one another– allowing them to produce more complex dynamics of musical meaning. This improvising collective exists only so long as the individual improvisers work to constrain one another, and allow the work of others to constrain them.

**FIGURE 4:**
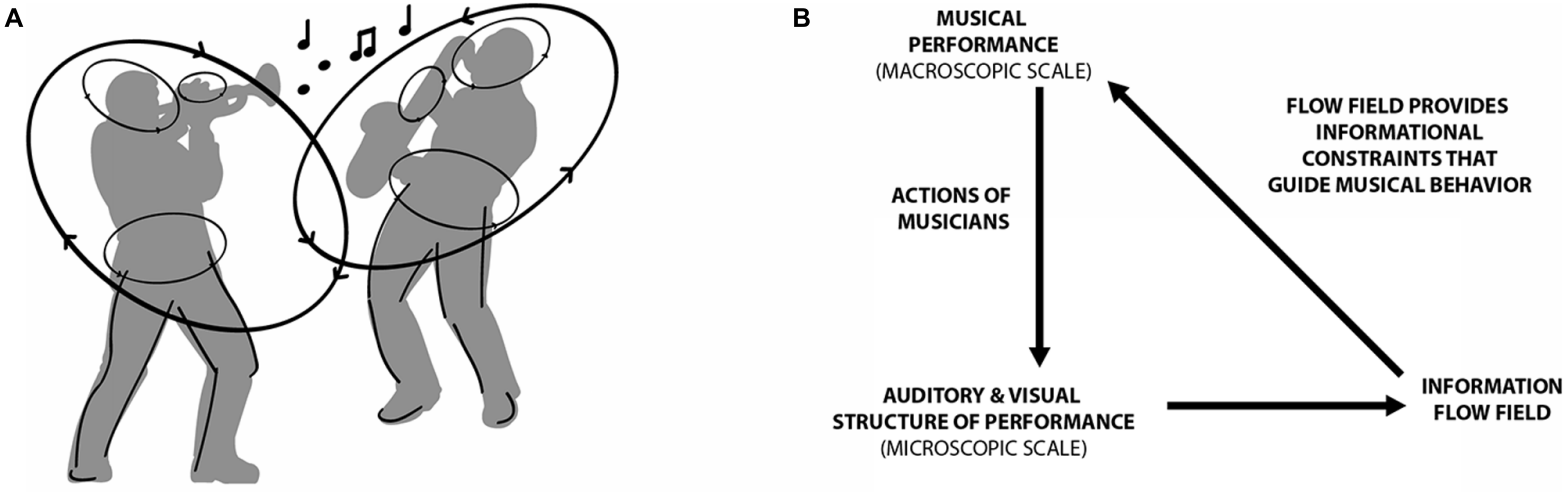
**Macroscopic and microscopic interaction involved in musical improvisation. (A)** Structural changes in visual and auditory information about co-performers actions at both local and global levels serve to constrain musical produce. **(B)** Adapted from Kugler and Turvey (1987), Illustrates the interaction between the micro and macroscopic scales, here the flow field of information refers to a the time-evolving structures of sound and light that are informative about current, future and past actions of the musicians as a group.

Instead of being concerned with the infinite possibilities available for musical expression in improvisatory performance, one can investigate how continuous information about the co-performer’s actions constrains, or limits, the range of expression a musician will produce when engaged in such coordination. As illustrated in **Figure 4**, the macroscopic musical performance that emerges during improvisation is both a result of the action as well as induces structure in auditory and visual information that further shapes the performances of the musicians. **Figures 4A** illustrates how the strength and relative phase of inter-musician coordination at the microscopic levels of the fingers, waist, or head movements generates visual and auditory structure, and this structure is then detected by the co-performer and thus constrains their musical performance (**Figures 4B**).

The application of cross wavelet spectral analysis to quantify coordination at both macro and microscopic spatiotemporal scales is just one step toward understanding how and when musicians detect and exploit multi-modal information about co-performers while improvising. Of course, attempting to understand behavior as it is situated within the context of the brain, body and environment introduces substantial variation. Yet these methods for analyzing complex dynamical systems allow for enlightening observations of how informational changes within even quite complex performance contexts affect the dynamics that emerge.

## Conclusion

We have proposed new ways of investigating how musicians are able to spontaneously coordinate their musical movements to produce something never seen or heard before. The methods of complex dynamical systems introduce new tools with which to investigate this impressive ability for novelty, and suggest new possibilities for experimental investigation. It is important to recognize that these patterns of movement coordination might be considered merely an artifact or result of some cognitive ability, such as the conceptual knowledge base described by Berkowitz. Yet substantive accounts of the supposed neurocognitive structures that serve as the substrates for this conceptual knowledge are remarkably thin on the ground. And, increasingly, there is reason to think that other approaches may prove fruitful. Van Orden et al. (2012) tells the story of Mike the headless chicken, who despite an accidental beheading, lived and grew for 18 months, sustained by meals administered through an eye dropper. Not only did Mike live, but was able to demonstrate the same range of body movement and coordination, without the help of the central nervous system. They claimed that Mike demonstrated how the brain might play a more “blue-collar role” in determining behavior than normally assumed, the body filling a more “white-collar” role (Van Orden et al., 2012, p. 1). And while this is not to suggest that improvising musicians are so many headless chickens, it is to say that there is much sophistication to be uncovered in the spatiotemporal processes of musicians’ bodily coordination. Understanding musicians as a self-organized system, coupled such that they constrain each other’s musical performance, provides an initial path for harnessing the complexity of what seems like an overwhelming task requiring magical skills. The theoretical and methodological approach outlined here allows us to see how musical meaning emerges from the coordinated, yet complex turn taking manifested in spontaneous musical expression.

## Conflict of Interest Statement

The authors declare that the research was conducted in the absence of any commercial or financial relationships that could be construed as a potential conflict of interest.
